# The Effect of Glycerol Monolaurate on Intestinal Health and Disease Resistance in Cage-Farmed Juvenile Pompano *Trachinotus ovatus*

**DOI:** 10.1155/2023/8580240

**Published:** 2023-04-24

**Authors:** Huaxing Lin, Beiping Tan, Qihui Yang

**Affiliations:** ^1^College of Fisheries, Guangdong Ocean University, Zhanjiang, 524088 Guangdong, China; ^2^Aquatic Animals Precision Nutrition and High Efficiency Feed Engineering Research Center of Guangdong Province, Zhanjiang, 524088 Guangdong, China; ^3^Guangdong Provincial Key Lab of Pathogenic Biology and Epidemiology for Aquatic Economic Animals, Zhanjiang, 524088 Guangdong, China

## Abstract

This research studied the effects of glycerol monolaurate (GML) to diets on the digestive capacity, intestinal structure, intestinal microbiota, and disease resistance for juvenile pompano *Trachinotus ovatus* (mean weight = 14.00 ± 0.70 g). *T. ovatus* were, respectively, fed six diets containing 0.00, 0.05, 0.10, 0.15, 0.20, and 0.25% GML for 56 days. The highest weight gain rate was observed in the 0.15% GML group. In the intestine, amylase activities in the 0.10, 0.15, 0.20, and 0.25% GML groups were significantly increased, compared with 0.00% GML group (*P* < 0.05). Lipase activities in the 0.10 and 0.15% GML groups were significantly increased (*P* < 0.05). Similar significant elevations in the protease activities were also found in the 0.10, 0.15, and 0.20% GML groups (*P* < 0.05). Amylase activities were significantly higher in the 0.10, 0.15, 0.20, and 0.25% GML groups than that in the 0.00% GML group (*P* < 0.05). Villus lengths (VL) and muscle thicknesses (MT) of the 0.05, 0.10, 0.15, and 0.20% GML groups were significantly enhanced, and the villus widths (VW) in the 0.05, 0.10, and 0.15% groups were significantly increased (*P* < 0.05). Additionally, 0.15% GML significantly improved the intestinal immunity by upregulating interleukin 10 (*il-10*), increasing beneficial bacteria abundances (e.g., *Vibrio*, *Pseudomonas*, and *Cetobacterium*), downregulating nuclear factor kappa b (*nf-κb*) and interleukin 8 (*il-8*), and decreasing harmful bacteria abundances (e.g., *Brevinema* and *Acinetobacter*) (*P* < 0.05). After challenge test, GML significantly increased the survival rate (80%–96%) (*P* < 0.05). In addition, ACP and AKP activities in the GML-supplemented groups were significantly higher than those in the 0.00% GML group, and LZM activity was significantly higher in the 0.05, 0.10, 0.15, and 0.20% GML groups than that in the 0.00% GML group (*P* < 0.05). In summary, 0.15% GML significantly promoted the intestinal digestibility, improved the intestinal microflora, regulated intestinal immune-related genes, and increased resistance to *V. parahaemolyticus* of juvenile pompano *T. ovatus*.

## 1. Introduction

Pompano *Trachinotus ovatus*, a carnivorous fish, has many advantages such as fast growth, tender meat, tasty taste, and moderate price [1]. In 2020, *T. ovatus* production in China exceeded 120,000 tones, making it a promising candidate [2]. However, as negative impacts arise, such as increasing scale and intensification of aquaculture and the pollution of the aquaculture environment, *T*. *ovatus* diseases occur frequently and are becoming increasingly serious [3]. Diseases have caused great economic losses to *T*. *ovatus* aquaculture production and seriously hindered the development of the aquaculture industry [4]. Improving or maintaining the animal's immunity of this fish plays a vital role and is the foundation of disease resistance, which is related to nutrition and feeding [5].

Disease outbreaks are a key limiting factor for the aquaculture industry and lead to the use of a large number of chemicals [6]. Antibiotics play a vital role in improving animal performance and health, but problems connected with their use, such as antibiotic residues, increased bacterial resistance, reduced immunity due to long-term use, and secondary infections, have caused great concern [7]. Therefore, the search for feed additives that can replace antibiotics and improve the immunity of aquatic animals is the focus of many researchers. Medium-chain fatty acids (MCFAs) are a class of energy substances with specific physiological functions that can be developed as feed additives as an alternative to antibiotics [8]. National and international researchers have found that MCFA monoglycerides, particularly glycerol monolaurate (GML), have antipathogenic properties [9–11].

GML, easily digested and well absorbed, is a typical fatty acid glyceride of the MCFA monoglycerides that has strong antibacterial properties [8, 12]. GML is used to accelerate growth, improve feed conversion rates, and enhance disease resistance for livestock and poultry [13]. Moreover, GML is directly absorbed by the intestinal epithelium. GML provides rapid energy to the intestinal epithelium, increases the height of the intestinal villi, improves digestion and absorption, and maintains the integrity of the animal's intestine [14]. In addition, GML can inhibit harmful bacteria and adjust and stabilize the balance of the intestinal microflora, thereby affecting the performance of animals [15]. A study showed that mice fed GML were able to significantly increase the abundance of beneficial intestinal bacteria and improve intestinal metabolism [16]. To date, studies have revealed that GML has growth-promoting and immunomodulatory effects on poultry [17, 18].

In our previous experiments, it was found that GML can remarkably improve the fat metabolism of pompano *T*. *ovatus*, thus affecting the growth performance [2]. Based on the nutritional properties of GML, an investigation into the relationship between GML on the growth and immune capacity of pompano *T. ovatus* was further explored. Therefore, we studied the effects of GML on digestibility, intestinal structure, intestinal microbiota, and disease resistance for pompano *T*. *ovatus*.

## 2. Materials and Methods

### 2.1. Animals and Diet Preparation

For all procedures relating to live animals, approval has been authorized by the Institutional Animal Care and Use Committee of the Guangdong Ocean University (ID GDOU-AEWC-20180063).

The experiment was conducted at an experimental site in Zhanjiang, Guangdong, China. Juvenile *T*. *ovatus* was procured from a seedling farm in Hainan Province for this investigation. The juvenile *T*. *ovatus* was acclimated to experimental conditions for two weeks.

Six isonitrogenous and isolipidic diets were randomly prepared: the basal diet with 0 (0.00%), 500 (0.05%), 1000 (0.10%), 1500 (0.15%), 2000 (0.20%), and 2500 (0.25%) mg/kg of GML. All raw materials are crushed, mixed, added with oil and water, and then granulated (2.5 mm in diameter) with a twin screw extruder (F-75; South China University of Technology, Guangzhou, Guangdong, China). After natural drying to about 10% moisture, they are stored at -20°C.

Fish acclimatization, feed and raising conditions, and diet preparation (Supplementary Table S1) are detailed in the supplementary material.

### 2.2. Sample Collection and Analyses

After 8 weeks, the fish for each floating cage were sampled, and relevant growth indicators were counted, including weight gain rate (WGR), specific growth rate (SGR), feed coefficient rate (FCR), and survival rate (SR). Afterwards, the serum of three fish for each floating cage was obtained to analyze the relevant enzyme activity indicators, including alkaline phosphatase (AKP), acid phosphatase (ACP), and lysozyme (LZM) activities. Detailed descriptions are in the supplementary material.

The intestine samples of five fish for each floating cage were quickly collected, and part of them were stored for enzyme activity analysis, whereas the rest were stored in RNA-later [19]. Complete intestines of three fish for each floating cage are obtained and kept at -80°C for gut flora analysis [20].

### 2.3. Histological Morphology

Intestines of three fish for each floating cage were quickly removed and preserved in a 4% paraformaldehyde solution and analyzed histologically with hematoxylin-eosin (H&E) [21]. Intestinal sections were viewed under a light microscope and photographed, with random measurements of VL, VW, and MT.

### 2.4. Challenge Test

After initial sampling, the experimental fish were transported to the Donghai Island Breeding base of Guangdong Ocean University to be stored in tanks (0.5 m^3^) for temporary rearing. During transport, the “closed transport with circulating water” method was adopted, including fish-holding techniques, low-temperature dormancy techniques, fish transport equipment, and environmental control techniques. The fish in each group exhibited 0% mortality during transport and were fed with each group of feed during transient rearing, respectively.

*V*. *parahaemolyticus* was obtained from Guangdong Provincial Key Laboratory of Pathogenic Biology and Epidemiology for Aquatic Economic Animals and activated twice [22]. A pretest determined a semilethal concentration (LD_50_, 7 d) of 2 × 10^8^ cfu/ml. A challenge test was conducted in triplicate with 10 fish per replicate. All fish were injected intraperitoneally with a bacterial suspension (0.2 ml). No diets were provided to these animals during the trial. Mortality was observed in each tank at 7 d, and SR was determined. Serum was obtained similarly to “2.2 sample collection and analyses”. Detailed descriptions are in the supplementary material.

### 2.5. Intestinal Microbiota Sequencing Analysis

Microbial DNA was obtained with HiPure Soil DNA Kit (or HiPure Fecal DNA Kit) (Magen, Guangzhou, China). PCR amplification was performed using primers (27F: 5′-AGRGTTYGATYMTGGCTCAG-3′; 1492R: 5′-RGYTACCTTGTTACGACTT-3′) to obtain full-length 16S rDNA. Amplicons were assessed with a 2% agarose gel and purified employing the Axy-Prep DNA Gel Extraction Kit (Axy-gen Biosciences, Union City, CA, USA). The purified PCR products were sequenced in high throughput (Illumina Hiseq2500 sequencing system). After sequencing was completed, the raw reads were screened as follows [23]: (1) reads filtering, (2) reads assembly, (3) raw tag filtering, and (4) clustering and chimera removal. Through the above 4 steps of processing, a valid label was finally obtained for further analysis. Sequences of the most abundant tags were chosen as representative of each cluster. Detailed descriptions are in the supplementary material.

### 2.6. Real-Time PCR Analysis

RNA extraction, cDNA synthesis, and relative mRNA expression calculations were carried out as previously published [2, 24]. Detailed descriptions are in the supplementary material. The PCR primers were shown in Table 1 with *nf-κb*, *il-10*, *il-8*, *il-1β*, and *β-actin*, respectively.

### 2.7. Statistical Analysis

All data were analyzed by one-way analysis of variance (ANOVA) using SPSS 21.0 (SPSS Inc., Chicago, IL, USA), followed by Duncan's multiple range test to determine significant differences between the groups (*P* < 0.05).

## 3. Results

### 3.1. Effect of GML on Growth Performance

The juvenile pompano *T. ovatus* was fed 0 (0.00%), 500 (0.05%), 1000 (0.10%), 1500 (0.15%), 2000 (0.20%), and 2500 (0.25%) mg/kg of GML for 56 days. WGR were significantly higher in the 0.10% and 0.15% GML groups than that in the other groups (*P* < 0.05). The highest WGR was observed in the 0.15% GML group (*P* < 0.05; Supplementary Table S2).

### 3.2. Effects of GML on Digestive Enzyme Activities

In the intestine, amylase activities were significantly higher in the 0.10, 0.15, 0.20, and 0.25% GML groups than that in the 0.00% GML group (*P* < 0.05; Table 2). Lipase activities were significantly higher in the 0.10 and 0.15% GML groups than that in the 0.00% GML group (*P* < 0.05). Protease activities were significantly higher in the 0.10, 0.15, and 0.20% groups than that in the 0.00% group (*P* < 0.05).

### 3.3. Effects of GML on Histological Morphology

**Figure 1:**
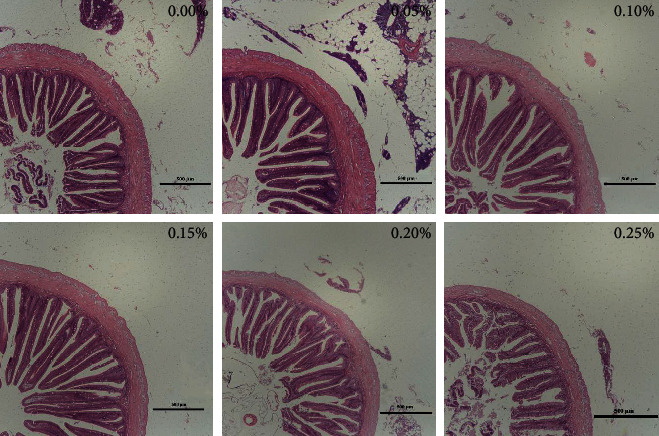
Light microscopy of the hindgut intestine morphology for juvenile pompano *T. ovatus* fed diets with GML (H&E staining).

In Table 3 and Figure 1, VL, VW, and MT were all significantly higher in the 0.15% GML group than that in the 0.00% GML group (*P* < 0.05). The VL in the 0.05, 0.10, 0.15, and 0.20% GML groups were significantly higher than those in the 0.00% GML group (*P* < 0.05), whereas the difference between the 0.25 and 0.00% GML groups was not significant. The VW in the 0.05, 0.10, 0.15, and 0.20% GML groups were significantly higher than those in the 0.00% GML group (*P* < 0.05). The 0.15% GML group had the widest VW. MT corresponded to the trend in VW. The MT values in the 0.05, 0.10, and 0.15% GML groups were significantly higher than those in the 0.00% GML group (*P* < 0.05).

### 3.4. Effects of GML on the Intestinal Bacterial Community

**Figure 2:**
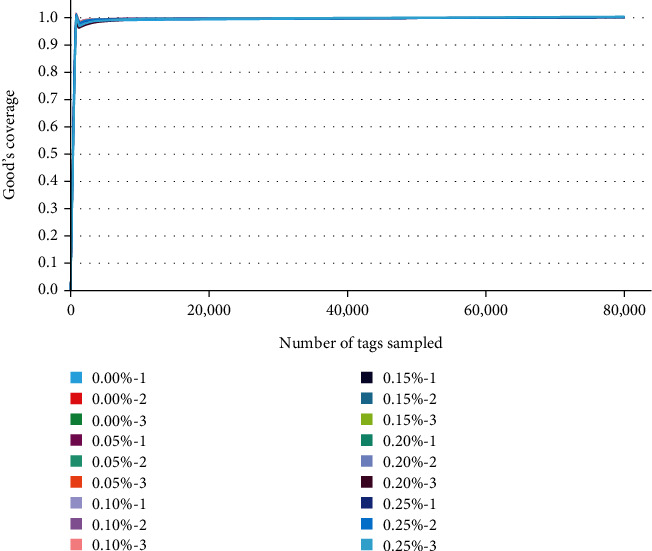
Sequencing depth coverage curve.

In Figure 2, the coverage of the samples was greater than 99% in all 18 samples, indicating that the bacteria were largely identified. No significant differences were observed in Ace, Chao1, Simpson, and Shannon among all groups (*P* > 0.05; Table 4).

**Figure 3:**
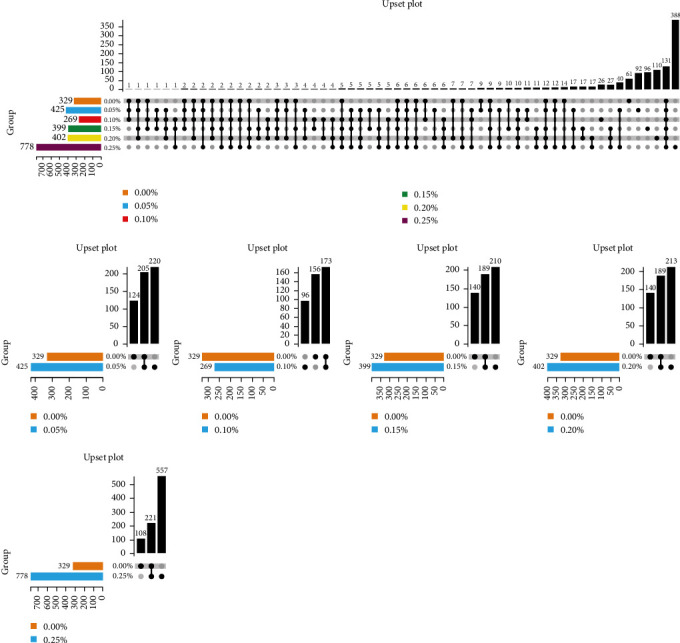
Upset diagram for unique and shared OTUs. The left bar is the total number of OTUs contained in each original dataset; the lower intersection point indicates the name of the group corresponding to the left-hand side. The intersection between the corresponding groups is indicated by the vertical realization of the connection between the points; the upper bar indicates the number of intersection elements in this intersection case.

In Figure 3, the numbers of intestinal unique operational taxonomic units (OTUs) were 220, 96, 210, 213, and 557 in the 0.05, 0.10, 0.15, 0.20, and 0.25% GML groups, respectively. The numbers of shared OTUs were 205, 173, 189, 189, and 221, respectively, compared with the 0.00% GML group. A total of 131 shared OTUs were found in the six experimental sample groups. The unique OTUs in the 0.00, 0.05, 0.10, 0.15, 0.20, and 0.25% GML groups were 61, 92, 26, 96, 110, and 388, respectively.

**Figure 4:**
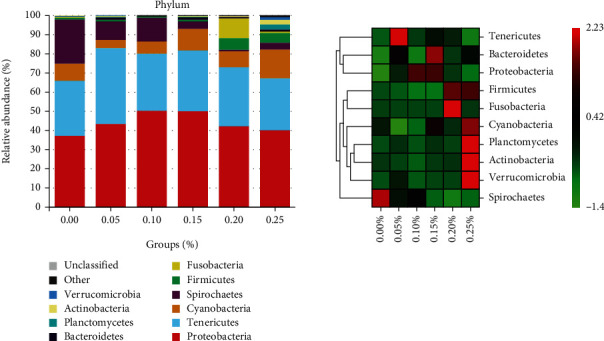
At phylum level, relative abundance and heat map analysis of intestinal microbiota for juvenile *T. ovatus* with different dietary.

**Figure 5:**
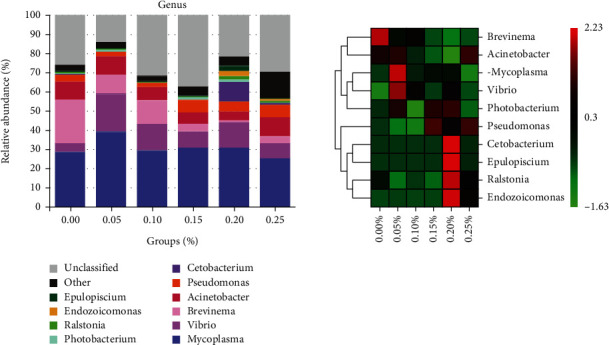
At genus level, relative abundance and heat map analysis of intestinal microbiota for juvenile *T. ovatus* with different dietary.

At the phylum level, ten dominant phyla were certified, including Proteobacteria, Tenericutes, *Cyanobacteria*, Spirochaetes, *Bacteroidetes*, *Firmicutes*, *Fusobacteria*, *Planctomycetes*, *Actinobacteria*, and *Verrucomicrobia* (Figure 4). In the intestinal microbiota, the abundance of Proteobacteria and Tenericutes was primarily dominant; their sum was above 60% in all groups. Proteobacteria abundance was higher in all experimental groups than that in the control group. In the 0.00% GML group, Spirochaetes abundant in 22.71% of the intestinal microbiota were significantly higher than that in the other groups (*P* < 0.05). The Spirochaetes phylum showed a gradual decrease in abundance as the supplemented GML was increased. At the genus level, the intestinal bacterial flora of juvenile *T*. *ovatus* comprised *Mycoplasma*, *Vibrio*, *Brevinema*, *Acinetobacter*, *Pseudomonas*, and *Cetobacterium*, along with some genera that could not be identified (Figure 5). *Vibrio* abundance in the GML-supplemented groups was higher than that in the 0.00% GML group. *Brevinema* abundance was 22.71% in the 0.00% GML group, significantly higher than that in the GML-supplemented groups (*P* < 0.05). With increasing GML rate, the abundance of *Brevinema* showed a gradual decrease, whereas that of *Acinetobacter* showed a decrease followed by an increase. The percentages of *Pseudomonas* in the 0.00, 0.05, 0.10, 0.15, 0.20, and 0.25% GML groups were 3.57, 2.50, 2.09, 6.72, 5.21, and 6.60%, respectively.

**Figure 6:**
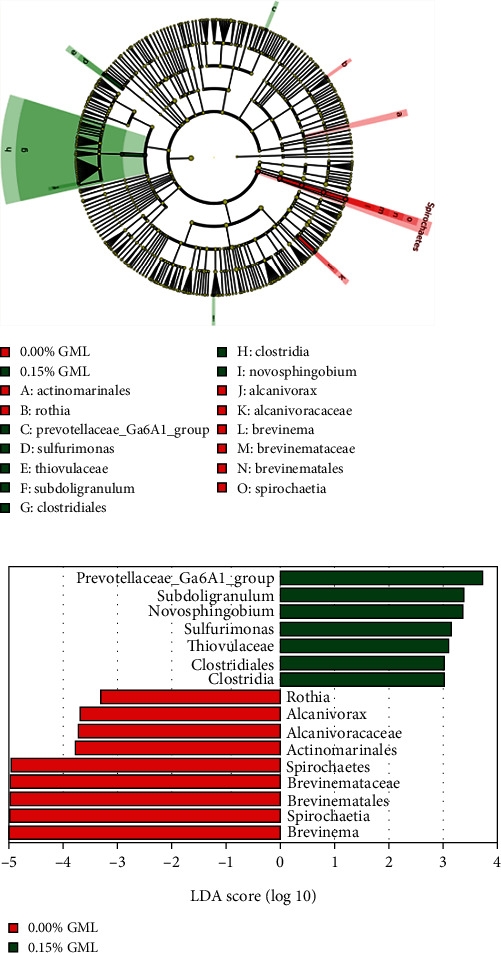
Linear discriminant analysis (LDA) score threshold greater than 3 was presented to demonstrate the variation in the relative abundance of the intestine microbial communities between pompano *T. ovatus* fed diet 0.00% GML and 0.15% GML.

In Figure 6, the linear discriminant analysis (LDA) effect size (Lefse) package was applied to identify the relative abundance of microbial taxa that differed between the 0.15% GML group and the control group. The LDA score threshold was set to >3. The linear discriminant analysis (LDA) shows that the LDA score of Lefse in the 0.15% GML group revealed an increase in seven taxa and a decrease in nine taxa, compared with the 0.00% GML group.

### 3.5. Effects of GML on Intestinal Immunity-Related Gene Expression

**Figure 7:**
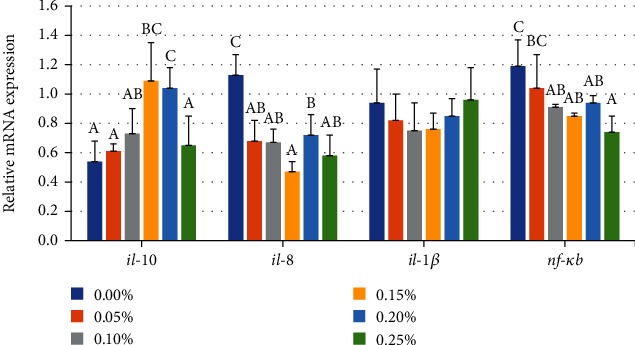
Relative expression levels of immune-related gene in intestine for juvenile *T. ovatus*. Data were expressed as means ± SEM. Different letters above a bar are statistically significant different among treatments (*P* < 0.05).

In Figure 7, the mRNA levels of *il-10* decreased first and then significantly increased. Compared with the 0.00% GML group, *il-10* expression level was significantly upregulated in the 0.15 and 0.20% GML groups (*P* < 0.05). *il-8* expression level was significantly downregulated with GML supplementation (*P* < 0.05), with the lowest *il-8* expression that was found in the 0.15% GML group. *nf-κb* expression levels were significantly lower in the 0.10, 0.15, 0.20, and 0.25% GML groups than that in the 0.00% GML group (*P* < 0.05). No significant differences were found in *il-1β* expression levels (*P* > 0.05).

### 3.6. Effects of GML on Challenge Test with *V*. *parahaemolyticus*

**Figure 8:**
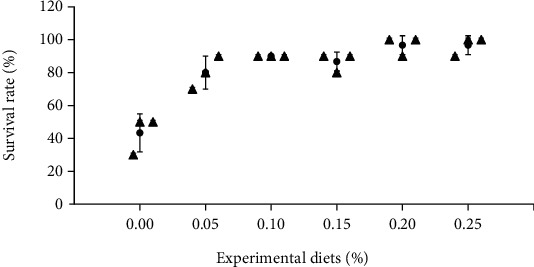
Effects of GML on the survival rate after *V. parahaemolyticus* (2 × 10^8^ cfu/ml) infection of juvenile *T. ovatus* at 7th day. Data were expressed as the mean ± SEM. Values not sharing a common superscript were significantly different (*P* < 0.05).

In Figure 8, after the challenge test of *V*. *parahaemolyticus*, dietary supplementation with GML significantly increased the SR (*P* < 0.05) compared with the 0.00% GML group. The SR values in the 0.05, 0.10, 0.15, 0.20, and 0.25% GML groups were significantly higher than that in the 0.00% GML (*P* < 0.05).

After the challenge test, ACP and AKP activities in the GML-supplemented groups were significantly higher than those in the 0.00% GML group (*P* < 0.05; Table 5). LZM activity was significantly higher in the 0.05, 0.10, 0.15, and 0.20% GML groups than that in the 0.00% GML group (*P* < 0.05).

## 4. Discussion

The effect of GML on animal health and growth has caused widespread concern [25, 26]. Studies has shown that a diet supplemented with 0.075% GML significantly improved WGR and SGR of *Danio rerio* [15] and *L*. *croceus* [27]. In addition, 0.07 and 0.105% GML significantly improved WGR and improved the intestinal microbiota of *L*. *vannamei* [28]. These results are in line with those obtained in previous research reported on the growth-promoting potential of GML [29]. In the present study, a similar conclusion was reached; 0.15% GML in the diet significantly improved the growth.

Animal growth is closely related to feeding utilization, which is influenced by digestion and absorption capacities [30]. Digestive enzymes speed up the breakdown and utilization of the corresponding nutrients by the intestines, thereby improving feed utilization and promoting animal growth. A study has shown that GML could penetrate deep into the intestinal tract and greatly affect digestibility and nutrient utilization [31]. In *L*. *vannamei*, GML improved protein digestibility by increasing lipase and protease activities [28]. In addition, MCFAs and the corresponding glycerides increased chymotrypsin activity and protein digestibility, thereby affecting growth similar to that of Atlantic salmon (*Salmo salar L.*) [32]. Similar conclusions were reached in this study. Thus, GML can significantly enhance the intestinal digestibility of *T*. *ovatus*.

The intestine is the most important organ for digestion and absorption in animals, and a healthy intestinal structure is a basis for digestive and absorption functions [33]. The VL, VW, and MT of the intestine are important indicators of the digestive and absorptive function and the health of the intestinal mucosal tissue structure. The height and length of the villus are significantly correlated with the number of mature cells. Only mature villi can absorb nutrients. The longer the villus length is, the larger the nutrient absorption area will be. In the present study, GML significantly increased the VL, VW, and MT, and these results were direct responses to the ability of the GML to improve intestinal digestion. GML improved the morphological structure of the intestine, because it was used directly by the intestinal epithelial cells when absorbed by the villous epithelium as an energy supply substance. GML promoted the growth of the villous epithelial cells [14]. Therefore, GML improves intestinal villi growth in juvenile *T*. *ovatus*, contributing to the intestinal morphological integrity and absorption of nutrients.

In addition, as an essential immune organ in animals, the intestine protects against external pathogens. GML has good anti-inflammatory effects, alleviates the body's inflammatory response through multiple pathways, and participates in the body's immune regulation [34]. One of the main pathways affecting immunity is the activation of *nf-κb*, an inevitable central regulator of the inflammatory response involved in the signaling pathways of most intrinsic immune receptors [35]. In the present study, GML significantly decreased the *nf-κb* expression level, which was in agreement with Kong et al. [36]. The *nf-κb* is involved in *il-8* transcription and influences its regulation. The *il-8* is one of the proinflammatory cellular factors with a widespread role in promoting inflammation [37]. In addition, GML could regulate *il-10* expression level, an inflammatory anticellular factor [15]. The *il-10* is the main anti-inflammatory cytokine in fish and inhibits the overactivation of the immune response [38]. In the present study, *il-10* gene upregulation and *il-8* gene downregulation were the most significant in the 0.15% GML group. Therefore, this result possibly showed that GML supplementation might trigger specific immunological networks of juvenile pompano *T*. *ovatus*, and further research is needed.

The fish intestinal microbiota is a dynamic community of aerobic, partly anaerobic, and anaerobic bacteria. This community is a special dynamic environment known as the intestinal “island” microbiota. A balanced intestinal microecology is essential for healthy fish growth, and the balance of the microecology needs to be maintained by a wide range of beneficial intestinal bacteria. GML has a good antibacterial effect and helps stabilize the balance of the animals' intestinal microbiota [39]. In the present study, an alpha diversity analysis revealed no significant differences, indicating that the microbial diversity of juvenile pompano *T. ovatus* fed with GML was not separated.

At the phylum level, the dominant intestinal microbiota in juvenile *T*. *ovatus* included Proteobacteria and Tenericutes, in agreement with existing research [40, 41]. The predominant beneficial microflora, such as Proteobacteria, *Firmicutes*, and *Bacteroidetes*, provided exogenous digestive enzymes that can dissolve food into small molecules [42], thereby enhancing absorption and utilization in the fish gut [43]. In the present study, GML increased intestinal digestive enzyme activity, possibly associated with an increase in beneficial intestinal bacterial populations. In addition, Tenericutes have a beneficial role in fish growth and the suppression of pathogenic bacteria [44]. However, an increase in the proportion of *Helicobacter pylori* leads to an imbalance in the intestinal microbiota and a decrease in animal immunity. At the genus level, *Vibrio* is reportedly a probiotic for aquatic animals, and some bacteriostatic and growth-promoting species exist, such as *Vibrio alginolyticus* [45]. *Pseudomonas* produces a range of compounds with a wide range of antifouling biological activities and enables the production of low-temperature proteases, which remain active in certain extreme environments [46]. However, in the present study, harmful bacteria, such as *Brevinema* [47] and *Acinetobacter* [48], existed in the intestine. *Brevinema* richness gradually decreased as GML increased, with the lowest level in the 0.15% GML group. A study indicated that in *Sparus aurata*, GML could increase the relatively abundance of positive bacteria, namely, *Lactobacillus* [29]. Therefore, GML is effective in inhibiting harmful bacteria in the juvenile *T*. *ovatus* gut. In addition, the 0.00 and 0.15% GML groups were also analyzed using LDA according to their growth. Compared with the 0.00% GML group, beneficial bacteria, such as *Sulfurimonas*, *Novosphingobium*, and *Subdoligranulum*, increased in the 0.15% GML group. By contrast, harmful bacteria, such as *Brevinema*, *Brevinematales*, *Brevinemaceae*, and *Acinetobacter*, decreased in the 0.00% GML group. Therefore, 0.15% GML can significantly promote intestinal health.

Available methods are considerably limited for conducting a comprehensive study on fish immunity and disease resistance. Thus, finding effective biomarkers of disease resistance in fish is difficult. Bacterial challenge experiments facilitate the assessment of the effectiveness of feeds in protecting against pathogens and are often employed as the final indicator of the fish health status following nutritional experiments [49]. *V*. *parahaemolyticus*, a Gram-negative bacterium, is one of the most serious pathogens in mariculture systems [50]. In the present study, GML could significantly enhance SR (80%-96%) in the *V*. *parahaemolyticus* challenge test. Moreover, after the challenge test, GML significantly improved serum immune enzyme activities (i.e., AKP, ACP, and LZM). These results may be due to the fact that GML can easily cross the cell wall and bind to the biofilm to exert its inhibitory effect on the pathogen [39]. Therefore, feed supplementation with GML significantly improved the disease resistance of juvenile *T*. *ovatus*.

## 5. Conclusion

In conclusion, the research indicated that GML significantly improved growth and intestinal health for juvenile pompano *T*. *ovatus*. In addition, 0.15% GML significantly increased serum immune enzyme activity, promoted the intestinal digestibility, improved the intestinal microflora, regulated intestinal immune-related genes, and increased resistance to *V. parahaemolyticus* of juvenile pompano *T. ovatus*.

## Figures and Tables

**Table 1:** Sequences of primers used for real-time quantitative PCR.

Gene name	Primer sequence (5′ −3′)	Reference
*nf-κb*	F-TGCGACAAAGTCCAGAAAGAT R-CTGAGGGTGGTAGGTGAAGGG	[51]
*il-8*	F-GAGAAGCCTGGGAATGGA R-GAGCCTCAGGGTCTAAGCA	[51]
*il-10*	F-CTCCAGACAGAAGACTCCAGCA R-GGAATCCCTCCACAAAACGAC	[52]
*il-1β*	F-CGGACTCGAACGTGGTCACATTC R-AATATGGAAGGCAACCGTGCTCAG	[53]
*β-actin*	F-TACGAGCTGCCTGACGGACA R-GGCTGTGATCTCCTTCTGC	[54]

Abbreviations: *nf-κb*: nuclear factor kappa b; *il-8*: interleukin 8; *il-10*: interleukin 10; *il-1β*: interleukin 1*β*.

**Table 2:** Effect of GML on amylase, lipase, and protease activities in the intestine for juvenile pompano *T*. *ovatus*.

Parameters	Experimental diets					
	0.00%	0.05%	0.10%	0.15%	0.20%	0.25%
Amylase (mIU/mg.pro)	237.98 ± 32.52^a^	273.50 ± 18.96^a^	375.66 ± 55.75^b^	427.51 ± 44.73^b^	439.01 ± 1.02^b^	374.30 ± 31.14^b^
Lipase (mU/mg.pro)	1028.18 ± 36.93^a^	1080.37 ± 61.58^a^	1358.07 ± 76.50^c^	1200.83 ± 27.53^b^	1077.84 ± 89.37^a^	1086.58 ± 44.19^a^
Protease (U/mg.pro)	3208.49 ± 68.95^a^	3509.33 ± 188.49^a^	5906.64 ± 280.18^c^	4895.82 ± 153.36^b^	4834.59 ± 159.60^b^	3391.23 ± 129.42^a^

Note: data are mean ± SEM (*n* = 3). Values in the same row with different superscripts represent a significant difference (*P* < 0.05).

**Table 3:** Effect of GML on hindgut intestine morphology for juvenile pompano *T*. *ovatus*.

Parameters	Experimental diets					
	0.00%	0.05%	0.10%	0.15%	0.20%	0.25%
VL (*μ*m)	860.64 ± 22.34^a^	958.38 ± 12.12^b^	973.78 ± 7.18^bc^	1025.84 ± 59.00^c^	996.96 ± 7.82^bc^	881.71 ± 22.55^a^
VW (*μ*m)	131.29 ± 3.13^a^	145.19 ± 5.19^b^	147.59 ± 3.60^b^	168.29 ± 1.27^c^	127.82 ± 2.33^a^	125.86 ± 1.69^a^
MT (*μ*m)	205.34 ± 3.63^a^	222.60 ± 5.33^b^	232.42 ± 4.19^b^	271.46 ± 9.01^d^	254.62 ± 5.04^c^	201.98 ± 7.22^a^

Note: data are mean ± SEM (*n* = 3). Values in the same row with different superscripts represent a significant difference (*P* < 0.05). VL: villus length; VW: villus width; MT: muscle thickness.

**Table 4:** Effect of GML on alpha diversity indices in the intestinal microbiota for juvenile pompano *T*. *ovatus*.

Parameters		Experimental diets					
		0.00%	0.05%	0.10%	0.15%	0.20%	0.25%
Richness estimators	Ace	312.75 ± 28.58	331.12 ± 22.52	333.94 ± 17.01	345.96 ± 1.40	347.46 ± 40.06	378.91 ± 31.20
	Chao1	318.34 ± 40.42	323.99 ± 20.84	330.11 ± 2.64	356.31 ± 17.59	386.18 ± 40.67	381.41 ± 34.87
Diversity estimators	Simpson	0.82 ± 0.03	0.81 ± 0.02	0.75 ± 0.06	0.76 ± 0.04	0.85 ± 0.07	0.86 ± 0.10
	Shannon	3.11 ± 0.18	3.08 ± 0.12	2.67 ± 0.33	3.56 ± 0.68	3.10 ± 0.77	4.03 ± 0.94

Note: data are mean ± SEM (*n* = 3).

**Table 5:** Effect of GML on serum immune parameters after challenge for juvenile pompano *T*. *ovatus*.

Parameters	Experimental diets					
	0.00%	0.05%	0.10%	0.15%	0.20%	0.25%
AKP (U/mL)	2.49 ± 0.51^a^	3.37 ± 0.42^b^	5.57 ± 0.62^d^	4.34 ± 0.20^c^	4.11 ± 0.09^bc^	3.46 ± 0.39^b^
ACP (U/mL)	2.59 ± 0.56^a^	5.18 ± 0.14^b^	8.03 ± 0.74^d^	6.59 ± 0.25^c^	5.66 ± 0.54^b^	5.11 ± 0.58^b^
LZM (U/mL)	0.99 ± 0.23^a^	1.36 ± 0.13^b^	1.39 ± 0.08^b^	1.49 ± 0.17^b^	2.19 ± 0.17^c^	1.28 ± 0.20^ab^

Note: data are mean ± SEM (*n* = 3). Values in the same row with different superscripts represent significant difference (*P* < 0.05). AKP: alkaline phosphatase; ACP: acid phosphatase; LZM: lysozyme.

## Data Availability

The data that support the findings of this study are available on request from the corresponding author. The data are not publicly available due to privacy or ethical restrictions.

## Supplementary Materials

Supplementary Materials Supplementary material to this article is as follows: 2.1 Animals and diet preparation. 2.2 Sample collection and analyses. 2.5 Intestinal microbiota sequencing analysis. 2.6 Real-time PCR analysis. Table S1: composition and nutrient levels of the experimental diets. Table S2: effects of GML on growth and feed utilization for juvenile *T. ovatus*.
