# Supplementary material for: The Effect of Glycerol Monolaurate on Intestinal Health and Disease Resistance in Cage-Farmed Juvenile Pompano Trachinotus ovatus
Source: Aquac Nutr. 2023 Apr 24;2023:8580240. doi: 10.1155/2023/8580240 (PMC10151148; doi:10.1155/2023/8580240)
Supplement: Supplementary Materials — Supplementary material to this article is as follows: 2.1 Animals and diet preparation. 2.2 Sample collection and analyses. 2.5 Intestinal microbiota sequencing analysis. 2.6 Real-time PCR analysis. Table S1: composition and nutrient levels of the experimental diets. Table S2: effects of GML on growth and feed utilization for juvenile T. ovatus. [file 8580240.f1.docx]

**SUPPLEMENTARY MATERIAL**

**2.1 Animals and diet preparation**

**2.1.1 Diet formulation and preparation**

The dietary formulations (41.00% crude protein) were shown in Table S1. Six iso-nitrogenous and iso-lipidic diets were randomly allocated to 18 floating cages (1 × 1 × 2 m^3^): the basal diet with 0 (0.00%), 500 (0.05%), 1000 (0.10%), 1500 (0.15%), 2000 (0.20%), 2500 (0.25%) mg/kg of GML (powdery, purity: 85%, Guangdong Lipid Science and Application Engineering Technology Research Centre, South China University of Technology) with three replicates each. Fish meal, chicken meal, soybean meal and were the main sources of protein in the diet.

All raw material was crushed through a 60-mesh sieve, mixed through a V-type vertical mixer (JS-14S; Zhejiang Zhengtai Electric Co., Ltd.), followed by adding with oil and water, and then pelleted (2.5 mm diameter) making use of a double screw extruder (F-75; South China University of Technology, Guangzhou Guangdong, China). After the prepared experimental feed was naturally dried to about 10% moisture, it was sealed in a vacuum-packed bag and stored at -20°C until it was fed.

**2.1.2 Experimental animals and breeding management**

The experiment was conducted at an experimental site in Zhanjiang, Guangdong, China. Juvenile *T*. *ovatus* was procured from a seedling farm in Hainan Province for this investigation. The juvenile *T*. *ovatus* were acclimated to experimental conditions for two weeks. After two weeks, the total number of experimental fish was 720, and there were 40 fish (initial body weight: 14.00 ± 0.70 g) in each floating cage, respectively. The 18 groups were randomly assigned to the six test diets, with three replicates each. All fishes were fed twice daily (7:00 and 18:00) to visual satiety for 56 days. During the experiment, water temperature ranged from 29.0 to 31.0℃ and the salinity was 24-26 ppt.

**2.2 Sample collection and analyses**

Fish samples were kept fast for 24 h before collection at the end of the trial and anesthetized with MS-222 (1:10,000). The fish samples of each experimental group were calculated and weighed, and it was determined the weight gain rate, survival rate, specific growth rate, and feed conversion rate. The formula for calculations is as follows

Weight gain rate (WGR, %) = 100 × (final body weight (g) − initial body weight (g)) / initial body weight (g);

Specific growth rate (SGR, %) = 100 × [ln (final body weight (g)) − ln (initial body weight (g))] / days;

Feed coefficient rate (FCR) = feed intake (g) / (final body weight (g) − initial body weight (g));

Survival rate (SR, %) = 100 × (final fish number / initial fish number);

Three fish samples of each floating cage were randomly selected and then stored at -20°C to detect the whole-body composition. Blood samples were gotten from the tail veins of seven randomly selected fish samples from each floating cage and then stored at 4°C for 12 h. After centrifuging (4000 ×g, 4°C, 10 min), the serum of fish samples was obtained and immediately preserved at -80°C for analyzing serum indicators. The enzyme activities (acid phosphatase (ACP), alkaline phosphatase (AKP) and lysozyme (LZM)) in serum were determined by using a detection kit (Nanjing Jian Cheng Bioengineering Institute, China).

The ingredients of the experimental diets and fish samples (crude protein, crude lipid, moisture and ash) were measured by using standard methods AOAC (Association of Official Analytical Chemists, 2005). The content of crude protein was assayed by means of Kjeldahl method (N × 6.25) and crude lipid was measured using the method of Soxhlet extraction. Moisture was measured by the way of drying whole-body samples at 105℃ until it reached a changeless weight. The content of ash was measured by a muffle furnace instrument burning at 550 ℃ for 12 h. Crude Fiber was measured using the method of Van Soest. Gross energy in diets was determined by using oxygen bomb calorimetry (IKA-C2000, Germany).

**2.5 Intestinal microbiota sequencing analysis**

Microbial DNA was obtained with HiPure Soil DNA Kit (or HiPure Fecal DNA Kit) (Magen, Guangzhou, China). The full-length 16S rDNA was amplified by PCR (95°C for 2 min, followed by 35 cycles of 95°C for 30s, 60°C for 45 s, and 72°C for 90s, with a final extension of 72°C for 10 min) using primers 27F: 5'-AGRGTTYGATYMTGGCTCAG-3';1492R: 5'-RGYTACCTTGTTACGACTT-3'. The PCR reaction was carried out in a 50μl reaction volume with Trans-Gen High-Fidelity PCR Super-Mix (TransGen Biotech, Beijing, China), 0.2μM forward and reverse primers, and 5 ng template DNA. Amplicons were assessed with a 2% agarose gel and purified employing the Axy-Prep DNA Gel Extraction Kit (Axy-gen Biosciences, Union City, CA, USA). High-throughput sequencing of the purified PCR products was performed with an Illumina Hiseq2500 sequencing system.

After sequencing was completed, the raw reads were screened as follows: (1) Reads filtering: reads containing more than 10% of unknown nucleotides and those with less than 50% of bases with quality (Q-value) >20 and merged of the paired-end clean reads as raw tags using FASTP (V0.18.0) (Chen et al., 2018). (2) Reads assembly: paired-end clean reads were merged as raw tags according to FLASH (version 1.2.11) (Magoc & Salzberg, 2011) with a minimum overlap of 10 bp and mismatch error rates of 2%. (3) Raw tag filtering: noisy sequences of original tags were filtered in specific filtering conditions to gain high-quality clean tags (Bokulich et al., 2013). The filtering requirements were as below: break raw tags from the first low-quality base site where the number of bases in the continuous low-quality value (the default quality threshold was ≤3) reached the set length (the default length was 3 bp); Then, filter tags whose continuous high-quality base length was less than 75% of the tag length. (4) Clustering and chimera removal: the clean tags were clustered into operational taxonomic units of ≥ 97% similarity with the utilization of the UPARSE pipeline (version 9.2.64) (Edgar, 2013). All chimeric tags were removed via the UCHIME algorithm (Edgar et al., 2011) and finally obtained effective tags for further analysis. The tag sequences possessing the highest abundance were picked as the representative sequences within each cluster.

**2.6 Real-time PCR analysis**

Total RNA from the intestine of three randomly selected fish from each floating cage was extracted with an RNA extraction kit (TransZol Up Plus RNA Kit, Beijing, China). PrimeScript™ RT-PCR Kit (TaKaRa, Kusatsu, Japan) was used to synthesize complementary DNA (cDNA) according to the manufacturers’ instructions. All the real-time PCR reactions were performed on a Roche LightCycler480II (Switzerland) using an SYBR @ Premix Ex TaqTMKit (Takara) and program consisted of one cycle at 95°C for 30 s followed by 40 cycles at 95°C for 5 s and 60°C for 30 s, a melting curve of 0.5°C increments from 65 to 95°C was performed. The amplification efficiency of all genes was approximately equal, ranging from 97.4% to 100.0%. The relative mRNA expressions were calculated using the 2^−ΔΔCT^ method.

**References**

1. AOAC. Official methods of analysis of the association of official analytical chemists international. (2005). 16th ed. Arlington, V A: Association of Official Analytical Chemists, 1995.

2. Chen, S., Zhou, Y., Chen, Y., & Gu, J. (2018). fastp: an ultra-fast all-in-one FASTQ preprocessor. *Bioinformatics*, 34(17), i884-i890. doi:10.1093/bioinformatics/bty560

3. Magoc, T., & Salzberg, S. L. (2011). FLASH: fast length adjustment of short reads to improve genome assemblies. *Bioinformatics*, 27(21), 2957-2963. doi:10.1093/bioinformatics/btr507

4. Bokulich, N. A., Subramanian, S., Faith, J. J., Gevers, D., Gordon, J. I., Knight, R., Mills, D. A., & Caporaso, J. G. (2013). Quality-filtering vastly improves diversity estimates from Illumina amplicon sequencing. *Nature methods*, 10(1), 57-59. doi:10.1038/nmeth.2276

5. Edgar, R. C. (2013). UPARSE: highly accurate OTU sequences from microbial amplicon reads. *Nature methods*, 10(10), 996-998. doi:10.1038/nmeth.2604

6. Edgar, R. C., Haas, B. J., Clemente, J. C., Quince, C., & Knight, R. (2011). UCHIME improves sensitivity and speed of chimera detection. *Bioinformatics*, 27(16), 2194-2200. doi:10.1093/bioinformatics/btr381

Table S1 Composition and nutrient levels of the experimental diets (dry matter basis, %)

| Ingredients | Diets | | | | | |
| --- | --- | --- | --- | --- | --- | --- |
|  | 0.00% | 0.05% | 0.10% | 0.15% | 0.20% | 0.25% |
| Fish meal | 25.00 | 25.00 | 25.00 | 25.00 | 25.00 | 25.00 |
| Chicken meal | 8.00 | 8.00 | 8.00 | 8.00 | 8.00 | 8.00 |
| Soybean meal | 12.00 | 12.00 | 12.00 | 12.00 | 12.00 | 12.00 |
| Peanut meal | 8.00 | 8.00 | 8.00 | 8.00 | 8.00 | 8.00 |
| Corn gluten meal | 8.00 | 8.00 | 8.00 | 8.00 | 8.00 | 8.00 |
| Wheat flour | 20.00 | 20.00 | 20.00 | 20.00 | 20.00 | 20.00 |
| CaH_2_PO_4_ | 1.50 | 1.50 | 1.50 | 1.50 | 1.50 | 1.50 |
| Vitamin C | 0.05 | 0.05 | 0.05 | 0.05 | 0.05 | 0.05 |
| Choline chloride | 0.30 | 0.30 | 0.30 | 0.30 | 0.30 | 0.30 |
| Soy lecithin | 0.50 | 0.50 | 0.50 | 0.50 | 0.50 | 0.50 |
| Fish oil | 1.50 | 1.50 | 1.50 | 1.50 | 1.50 | 1.50 |
| Soy oil | 1.50 | 1.50 | 1.50 | 1.50 | 1.50 | 1.50 |
| Vitamin premix ^a^ | 0.50 | 0.50 | 0.50 | 0.50 | 0.50 | 0.50 |
| Mineral premix ^b^ | 0.50 | 0.50 | 0.50 | 0.50 | 0.50 | 0.50 |
| DL-Methionine | 0.46 | 0.46 | 0.46 | 0.46 | 0.46 | 0.46 |
| L-Lysine | 0.72 | 0.72 | 0.72 | 0.72 | 0.72 | 0.72 |
| L-Threonine | 0.24 | 0.24 | 0.24 | 0.24 | 0.24 | 0.24 |
| L-Arginine | 0.26 | 0.26 | 0.26 | 0.26 | 0.26 | 0.26 |
| Microcrystalline cellulose | 10.97 | 10.92 | 10.87 | 10.82 | 10.77 | 10.72 |
| Glyceryl monolaurate | 0.00 | 0.05 | 0.10 | 0.15 | 0.20 | 0.25 |
| Total | 100.00 | 100.00 | 100.00 | 100.00 | 100.00 | 100.00 |
| Nutrient levels |  |  |  |  |  |  |
| Crude protein ^c^ | 40.67 | 40.73 | 39.86 | 40.52 | 40.55 | 39.93 |
| Crude lipid ^c^ | 12.18 | 12.56 | 12.26 | 12.35 | 12.41 | 12.37 |
| Crude ash ^c^ | 7.90 | 7.79 | 7.89 | 7.96 | 7.79 | 7.88 |

^a^ The vitamin premix: vitamin A, 500,000 IU/kg; vitamin D3, 100,000 IU/kg; vitamin E, 4,000 mg/kg; vitamin K3, 1,000 mg/kg; vitamin B1, 500 mg/kg; vitamin B2, 1,000 mg/kg; vitamin B6, 1,000 mg/kg; vitamin B12, 2.0 mg/kg; nicotinic acid, 4,000 mg/kg; D-calcium pantothenate, 2,000 mg/kg; folic acid, 100 mg/kg; biotin, 10.0 mg/kg; vitamin C, 15,000 mg/kg;

^b^ Mineral mixture: Fe, 10,000 mg/kg; Cu, 300 mg/kg; Zn, 5,000 mg/kg; Mn, 1,200 mg/kg; I, 80 mg/kg; Se, 30 mg/kg; Co, 20 mg/kg.

^c^ Crude protein, crude lipid and crude ash contents were measured values.

Table S2 Effects of GML on growth and feed utilization for juvenile *T*. *ovatus*

| Parameters | Experimental diets | | | | | |
| --- | --- | --- | --- | --- | --- | --- |
|  | 0.00% | 0.05% | 0.10% | 0.15% | 0.20% | 0.25% |
| WGR (%) | 306.71±14.42^ab^ | 309.90±9.99^b^ | 317.19±6.12^b^ | 349.14±11.92^c^ | 338.38±8.34^c^ | 289.98±6.53^a^ |
| SGR (%/d) | 2.50±0.06^ab^ | 2.52±0.05^ab^ | 2.55±0.03^b^ | 2.68±0.05^c^ | 2.64±0.04^c^ | 2.46±0.05^a^ |
| FCR | 1.73±0.06 | 1.78±0.06 | 1.69±0.04 | 1.64±0.09 | 1.69±0.09 | 1.79±0.03 |
| SR (%) | 99.17±1.44 | 98.33±1.44 | 98.33±2.89 | 97.50±2.50 | 98.33±2.89 | 98.33±2.89 |

Note: Data are mean ± *S.E.M*. (*n = 3*). Values in the same row with different superscripts represent significant difference (*P* < 0.05). WGR: weight gain rate; SGR: specific growth rate; FCR: feed conversion rate; SR: survival rate.
